# The Complete Mitochondrial Genome of *Triplophysa brevicauda* and the Analysis of Phylogeny and Selective Pressure Within Genus *Triplophysa*

**DOI:** 10.3390/genes17070734

**Published:** 2026-06-25

**Authors:** Zhenyu Xiong, Yuntao Tian, Chaoyang Luo, Xionghui Xu, Juan Zhang, Chengyao Yang, Xinya Ma, Yangsong Wu, Xianfu Li, Yuan Mu

**Affiliations:** 1 Center for Interdisciplinary Sciences, Dali University, Dali 671003, China; 15123901404@163.com (Z.X.); lixf@eastern-himalaya.cn (X.L.); 2 Institute of Eastern-Himalaya Biodiversity Research, Dali University, Dali 671003, China; 3 Key Laboratory of Ecological Adaptive Evolution and Conservation on Animals-Plants in Southwest Mountain Ecosystem of Yunnan Province Higher Institutes College, School of Life Sciences, Yunnan Normal University, Kunming 650500, China

**Keywords:** *Triplophysa brevicauda*, mitochondrial genome, phylogeny, selective pressure, positively selected sites

## Abstract

Background/Objectives: Phylogenetic relationships within *Triplophysa* remain unclear due to limited molecular data and complex traits. Mitochondrial genomes are robust markers for teleostean phylogeny and molecular evolution. This study aims to provide *Triplophysa brevicauda* mitochondrial genome and molecular evidence for its phylogenetic position and high-altitude adaptation. Methods: The complete mitochondrial genome of *T. brevicauda* was assembled via Illumina NovaSeq 6000 paired-end sequencing. Genomic characteristics were analyzed, with phylogenetic reconstruction based on complete mitochondrial genomes and selective pressure analyses for adaptive evolution. Results: The 16,570 bp circular genome encodes 13 PCGs, 22 tRNAs, two rRNAs, and a D-loop. All PCGs start with ATG (except *cox1*) and terminate with TAA, TAG, or incomplete *Triplophysa Phylogenetic* analysis supports four Triplophysa clades, with *T. brevicauda* sister to *Triplophysa nujiangensa*. Purifying selection is pervasive, with elevated non-synonymous rates in *atp8*, *nad2*, *nad4L*, *nad6*, and positive selection in *nad2*/*nad5*. Conclusions: Positive selection in *nad2* and *nad5* may relate to *T. brevicauda*’s plateau adaptation. This study provides valuable mitochondrial resources, supporting further research on *Triplophysa*’s phylogeny and high-altitude adaptive evolution.

## 1. Introduction

The Qinghai-Xizang Plateau and its adjacent basins have experienced distinctive geological evolution and formed extreme habitats. These environments are characterized by high altitude, persistent hypoxia and dramatic temperature changes. As a result, this region serves as a natural laboratory for studying organismal adaptation and ranks among Earth’s most significant biodiversity hotspots. Within this region, the cypriniform genus *Triplophysa* (family Nemacheilidae, subfamily Noemacheilinae) represents the most species-rich radiation of freshwater fishes, endemic to the plateau’s rivers, lakes, and subterranean drainages.

Taxonomic and phylogenetic resolution of the genus *Triplophysa* has remained challenging. Extensive intraspecific morphological plasticity and significant character overlap among closely related species hinder reliable identification using traditional morphological criteria. Consequently, morphology-based classifications have generated persistent controversy. The taxon initially described as *Cobitis microps* by Günther (1868) was reassigned to genus *Nemacheilus* [1]; Kessler (1874) divided the group into two genera based on swim bladder development [2]; Rendahl (1933) established the subgenus *Triplophysa* using male secondary sexual traits [3]; Bănărescu (1975) elevated it to the generic status [4]; Zhu (1989) subdivided the genus into two subgenera, *Triplophysa* and *Hedinichthys*, and revised the constituent species [5]; and Kottelat (2012) recognized both subgenera as distinct genera [6]. These conflicting schemes, founded on disparate morphological characters, have perpetuated taxonomic instability and continue to obscure species boundaries within *Triplophysa*. At present, the authoritative ichthyological databases NCBI Taxonomy (TaxID: 341122) and FishBase adopt a unified classification framework for *Triplophysa*. This genus is formally classified under the order Cypriniformes, suborder Cobitoidei and family Nemacheilidae, which has become the widely accepted taxonomic standard worldwide.

Molecular approaches have provided new insights into the phylogeny of *Triplophysa*. However, marker selection and analytical strategies yield discordant conclusions. Single-gene mitochondrial studies illustrate this inconsistency. In contrast, Zhao (2015) concatenated *cytb* data from 13 plateau and three cave-dwelling *Triplophysa* with cobitoid outgroups and found all cobitoid families to be monophyletic, with *Triplophysa* forming a robust clade [7]. Li et al. (2017) generated *cox1* barcodes for 244 individuals representing 33 species, strongly supporting both species delimitation and the monophyly of the genus. Mitogenome-scale analyses generally reinforce monophyly [8]. Wang et al. (2012) sequenced the complete mitochondrial genome of the hypogean species *Triplophysa rosa*, combined these data with published *cytb* sequences, and placed *T. rosa* basally within a monophyletic Qinghai-Xizang Plateau *Triplophysa* clade [9]. Wang et al. (2017) obtained the mitogenome of *Triplophysa xiangxiensis*; partitioned analyses positioned this cave lineage as sister to *T. rosa*, with the paired taxa nested within a maximally supported *Triplophysa* clade that formed the sister group to *Barbatula* [10]. Similar topologies were reported by Tang et al. (2013), Li et al. (2013), and Lei et al. (2016) upon inclusion of additional mitogenomes [11,12,13]. Expanding taxon sampling to 96 cobitoid species, Wang et al. (2016) recovered seven monophyletic families and, within Nemacheilidae, a *Triplophysa* clade supported by posterior probability = 1.00 and bootstrap = 100%. However, multilocus nuclear–mitochondrial datasets have produced incongruent results [14]. Šlechtová et al. (2024) concatenated six genes (*cytb*, *RAG1*, *IRBP2*, *MYH6*, *RH1*, *EGR3*) for 279 nemacheilid taxa and, although *Triplophysa* was placed within the “Northern Clade” of stone loaches, the genus was reconstructed as polyphyletic—split into two lineages separated by *Barbatula* and *Claea*—a result that sharply contrasts with the monophyly consistently inferred from mitogenomic data [15]. Collectively, while most molecular evidence supports *Triplophysa* monophyly, ongoing species discovery, recurrent taxonomic reassignment, and differential marker selection have prevented definitive resolution of its phylogenetic structure.

Mitochondria are the core organelles responsible for cellular energy metabolism, and the 13 protein-coding genes (PCGs) encoded by mitochondrial DNA constitute key components of the oxidative phosphorylation system [16]. Multiple studies have confirmed that mitochondrial genomes are reliable molecular markers for evaluating selective pressure and adaptive evolution in aquatic organisms [17,18]. For *Triplophysa* species inhabiting extreme plateau and cave environments, mitogenomic analyses have detected evident evolutionary differentiation and lineage-specific selection signals during their adaptive radiation [19]. Accordingly, using mitochondrial sequences to explore evolutionary selection and potential positive selection during the rapid diversification of plateau loaches is a feasible and widely adopted research strategy.

Characterized by maternal inheritance, structural stability, and a moderate evolutionary rate, the mitochondrial genome serves as an optimal marker for teleost phylogenetics, population demography, and studies of adaptive evolution [20,21]. Here, the complete mitochondrial genome sequence of *T. brevicauda*, endemic to the Qinghai-Xizang Plateau, was generated using high-throughput DNA Nanoball Sequencing (DNBSEQ) technology. Genome organization, nucleotide composition, codon usage bias, and selective regimes were systematically characterized, and the phylogenetic placement within *Triplophysa* was resolved by combining all publicly available complete mitochondrial genomes of *Triplophysa* species retrieved from the NCBI GenBank database. The resulting topology provides a fully resolved framework for future investigations into high-altitude loaches.

## 2. Materials and Methods

### 2.1. Sample Collection and Species Identification

An adult *Triplophysa* specimen was captured using seine net (Fisher Supply Co., Kunming, China) in the Yarlung Tsangpo River, Xizang Autonomous Region, China (93.7763° E, 30.9790° N, altitude 4713.47 m). Voucher material was deposited at Dali University under accession number [LSECDU-2026005], ensuring traceability and future reference. A pectoral fin clip was excised, fixed in 75% ethanol (Sinopharm Chemical Reagent Co., Ltd., Shanghai, China) at collection and stored at −80 °C until DNA isolation. This stringent preservation protocol guaranteed nucleic acid integrity for downstream molecular analyses. Morphometric identification followed “The Fauna of Cobitid Fishes in China” (ISBN: 753450788X). According to the diagnostic characteristics compiled in The Fauna of Cobitid Fishes in China, the specimen exhibited the key attributes of *T. brevicauda* and *T. microps*: lateral line that is complete or incomplete; caudal peduncle depth scarcely reduced toward the caudal fin; snout length shorter than the post-orbital head length; lips that are thick and smooth or deeply plicate; caudal fin margin emarginate; swim bladder posterior chamber reduced to a small membranous sac.

### 2.2. Mitochondrial Genome Sequencing, Assembly and Annotation

Fin clips were shipped to Personalbio Technology (Shanghai, China) for total DNA extraction with the TIANamp Genomic DNA Kit (Tiangen Biotech Co., Ltd., Beijing, China) following the manufacturer’s protocol; integrity was verified through 1% agarose electrophoresis. A 400 bp insert library was constructed from 300 ng high-molecular-weight DNA and sequenced on the Illumina NovaSeq 6000 platform (2 × 150 bp paired-end). Adapters and low-quality reads were trimmed with Cutadapt v1.16 (Marcel Martin, Uppsala, Sweden) to yield clean data [22]. Mitochondrial scaffolds were assembled iteratively with GetOrganelle v1.7.0+ (Jin JJ, Kunming, China) and NOVOPlasty v4.2 (Dierckxsens N, Brussels, Belgium) [23,24], via reference-guided assembly, using the mitogenome with GenBank accession number NC_073586.1 as the reference sequence, oriented against a reference mitogenome, and circularized. The complete molecule was annotated with MITOS2 v4.2 (Bernt M, Tübingen, Germany) using genetic code 05 (Vertebrate); following standard metazoan mitochondrial annotation procedures it annotated 13 protein-coding genes through sequence homology comparison, predicted 22 tRNA genes and 2 rRNA genes relying on conserved secondary structures, and determined the non-coding control region by analyzing intergenic regions between coding sequences; missing protein-coding genes were recovered by GenBlastG v1.3 (She R, Boston, MA, USA) [25,26], tRNAs were predicted with tRNAscan-SE v2.0 (Chan PP, Berkeley, CA, USA), and any remaining gaps were filled manually in MEGA 7 v7.505 to ensure full gene complement [27,28].

### 2.3. Sequence Analyses

A circular map was generated and refined with OGDRAW (https://chlorobox.mpimp-golm.mpg.de/OGDraw.html, accessed on 13 August 2025) to visualize gene order and transcriptional polarity [29]. Structural characterization, nucleotide composition, codon usage patterns and relative synonymous codon usage (RSCU) were quantified in PhyloSuite v1.2.2 using the “Mitogenome” and “5 Vertebrate mitochondrial” schemas [30]. AT- and GC-skew values were computed according to Perna & Kocher (1995): AT-skew = (A − T)/(A + T); GC-skew = (G − C)/(G + C) [31].

### 2.4. Phylogenetic Analyses

Identification was performed through a phylogenetic analysis of *cytb* nucleotide sequences. A total of 217 *Triplophysa* cytb sequences and one *Schistura* cytb sequence (outgroup) were retrieved from the NCBI GenBank and compiled (Table S1). The sequences were batch-imported and extracted with PhyloSuite v1.2.2 [30], aligned codon-wise under the vertebrate mitochondrial genetic code using MAFFT v7.505 [32], and refined with MACSE v2.07 [33]. Ambiguously aligned regions were removed with Gblocks v0.91b [34]. ModelFinder selected MG+F1X4 as the best-fit model under the Bayesian Information Criterion [35]. Maximum likelihood (ML) inference was conducted in IQ-TREE v1.6.12 with 1000 ultrafast bootstrap replicates to assess branch support [36]. The resulting topology was used to assign *Triplophysa* sp. to its sister lineage.

*cytb* phylogeny further recovers the individual within a well-supported clade (BS > 85%) with *T. brevicauda* (Figure S1). Consequently, the specimen is identified as *T. brevicauda*.

To clarify the phylogenetic position of the target species *Triplophysa* sp. within the genus, a tree was reconstructed based on complete mitochondrial genome sequences. The dataset consisted of 63 species of the genus *Triplophysa*, which served as ingroup taxa and representative closely related species covering multiple evolutionary clades of this genus for comprehensive comparison. Two species, *Schistura callichroma* and *Schistura fasciolata* (family Nemacheilidae, subfamily Noemacheilinae), were clearly defined as outgroup taxa. The genus *Schistura* has a moderate genetic divergence from *Triplophysa* and is widely accepted as a suitable outgroup for phylogenetic reconstruction of *Triplophysa* in previous ichthyological studies. All complete mitochondrial genome sequences were downloaded from NCBI GenBank (Table S2). Complete mitochondrial genome sequences were batch-extracted with PhyloSuite v1.2.2 [30], aligned codon-wise under the vertebrate mitochondrial genetic code using MAFFT v7.505 [32], and refined with MACSE v2.07 [33]. Ambiguous positions were removed with Gblocks v0.91b [34], and the filtered single-gene blocks were concatenated into a single supermatrix. ModelFinder selected GTR+F+I+I+R5 as the best-fit model under the Bayesian Information Criterion [35]. Maximum likelihood (ML) analysis was performed in IQ-TREE v1.6.12 with 1000 ultrafast bootstrap replicates [36].

Bayesian inference (BI) was performed using MrBayes 3.2.7 based on the same aligned complete mitochondrial genome. For BI analysis, we used the GTR+F+I+G4 substitution model. *S. callichroma* and *S. fasciolata* were designated as outgroups. Four simultaneous Markov chains (three heated and one cold) were run for 2,000,000 generations, with trees and parameters sampled every 1000 generations. The first 25% of sampled trees were discarded as burn-in, and the remaining trees were used to construct a 50% majority-rule consensus tree. Convergence of the MCMC chains was confirmed when the standard deviation of split frequencies dropped below 0.01 [37].

The resulting tree was visualized and annotated with iTOL v6 and finalized in Adobe Photoshop 2023 for publication [38].

### 2.5. Detection of Selective Pressure

Selection pressure was quantified by the ratio ω = *dN*/*dS*: ω > 1, =1 or <1 indicates positive, neutral or purifying selection, respectively. To estimate selective regimes acting on the 13 mitochondrial protein-coding genes (*atp6*, *atp8*, *cox1*, *cox2*, *cox3*, *cytb*, *nad1*, *nad2*, *nad3*, *nad4*, *nad4L*, *nad5*, *nad6*) across 63 *Triplophysa* species, codon-based analyses were implemented in CodeML (PAML 4.9) under the vertebrate mitochondrial genetic code (icode = 2) and F3 × 4 codon frequency model (CodonFreq = 2), with the previously inferred topology fixed [39].

Site models were employed to detect persistent positive selection. The nested pair M8a (ω ≤ 1) versus M8 (ω > 1 allowed) was compared through the likelihood ratio test (LRT); 2ΔL follows χ^2^ distribution. When the M8 model was significantly favored, sites with posterior probability ≥ 0.80 of ω > 1 were identified using Bayes empirical Bayes inference. Branch models assessed lineage-specific heterogeneity in selective pressure. The free ratio model (M1: independent ω per branch) was tested against the single-ratio model (M0: uniform ω) through LRT. Significant improvement of M1 over M0 indicates differential selection among lineages. Terminal-branch ω values were extracted; cases with dN = 0, dS = 0 or ω > 1 were discarded before calculating mean ω for each gene and for each species. Differences among genes and among species were evaluated through one-way ANOVA. Subsequently, Tukey’s honestly significant difference (Tukey HSD) post hoc multiple comparison test was applied to identify specific species pairs or gene groups that presented statistically significant differences.

For complementary detection of episodic diversifying selection, we used MEME and aBSREL algorithms on the Datamonkey 2.0 web server powered by HyPhy 2.5 [40,41,42,43]. With the aligned codon sequences and ML phylogenetic tree as inputs, MEME was used to identify episodically selected codon sites and aBSREL to screen branches under transient positive selection; all tests adopted a significance threshold of *p* < 0.05.

## 3. Results and Analyses

### 3.1. Genome Structure and Base Composition

**Figure 1:**
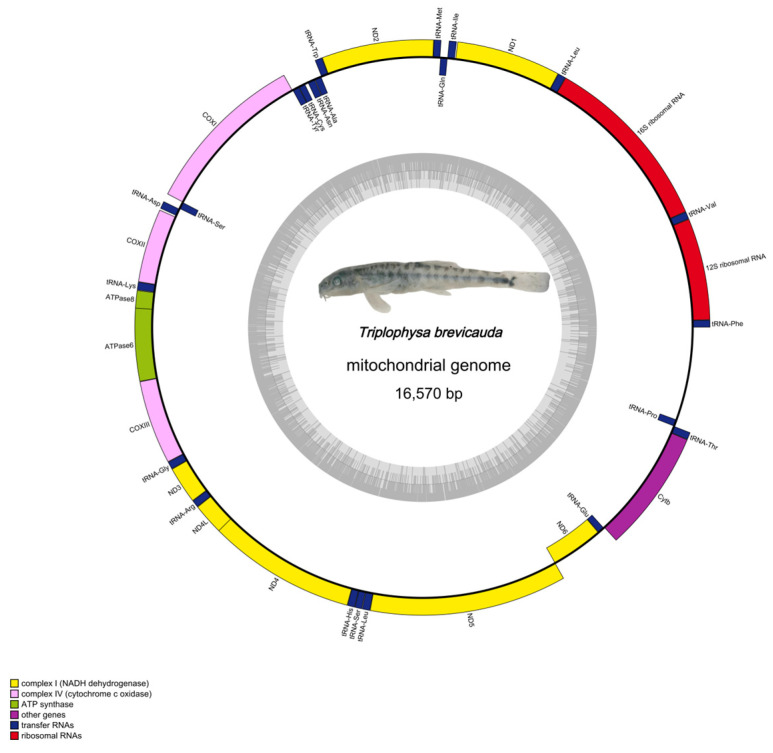
The circular map of the *T. brevicauda* mitogenome is shown. The outer and inner circles represent the L-strand and H-strand, respectively. The GC and AT contents are depicted by within the dark and light regions in the inner gray circle, respectively.

The complete mitochondrial genome of *T. brevicauda* is 16,570 bp and conforms to the canonical closed circular double-stranded conformation (Figure 1). Nucleotide composition is 28.22% A, 18.20% C, 25.55% G and 28.03% T, yielding a slight AT bias (56.25%). Consistent with other *Triplophysa* species, the molecule encodes 13 protein-coding genes (PCGs), 22 tRNAs, two rRNAs and a single control region (D-loop). Identified tRNAs range from 66 bp (*trnC*) to 76 bp (*trnK*). The 744 bp control region is interposed between *trnF* and *trnP* (Table S3).

### 3.2. Description of Protein-Coding Genes (PCGs)

The mitogenome of *T. brevicauda* include the canonical 37-gene set. Twenty-eight genes are encoded on the H-strand: *atp6*, *apt8*, *cox1*, *cox2*, *cox3*, *cytb*, *nad1*, *nad2*, *nad3*, *nad4*, *nad4l*, *nad5*, *rrnL*, *rrnS*, *trnD*, *trnF*, *trnG*, *trnH*, *trnI*, *trnK*, *trnL*, *trnM*, *trnR*, *trnS*, *trnT*, *trnV* and *trnW*. The L-strand encodes the remaining nine genes: *nad6*, *trnQ*, *trnA*, *trnN*, *trnC*, *trnY*, *trnS*, *trnE* and *trnP*. Among the protein-coding genes, ATG initiates all but cox1, which starts with GTG. Termination codons are TAA in six genes (*cox1*, *atp6*, *atp8*, *nad1*, *nad4L* and *nad5*), TAG in three (*nad2*, *nad3* and *nad6*), and an incomplete T in three others *(nad4*, *cox2* and *cytb*); *cox3* lacks a stop codon (Table S3).

Codon usage bias shapes translational efficiency and mRNA stability, thereby informing evolutionary patterning and phylogenetic signal. The 13 mitochondrial protein-coding genes of *T. brevicauda* comprise 3797 codons (Figure S2). Leucine (12.3%), isoleucine (9.8%), valine (7.5%) and phenylalanine (5.2%) are the most frequently encoded amino acids, while cysteine is the rarest (0.8%). Notably, TAA is the predominant termination codon.

### 3.3. Phylogenetic Relationship Analyses

**Figure 2:**
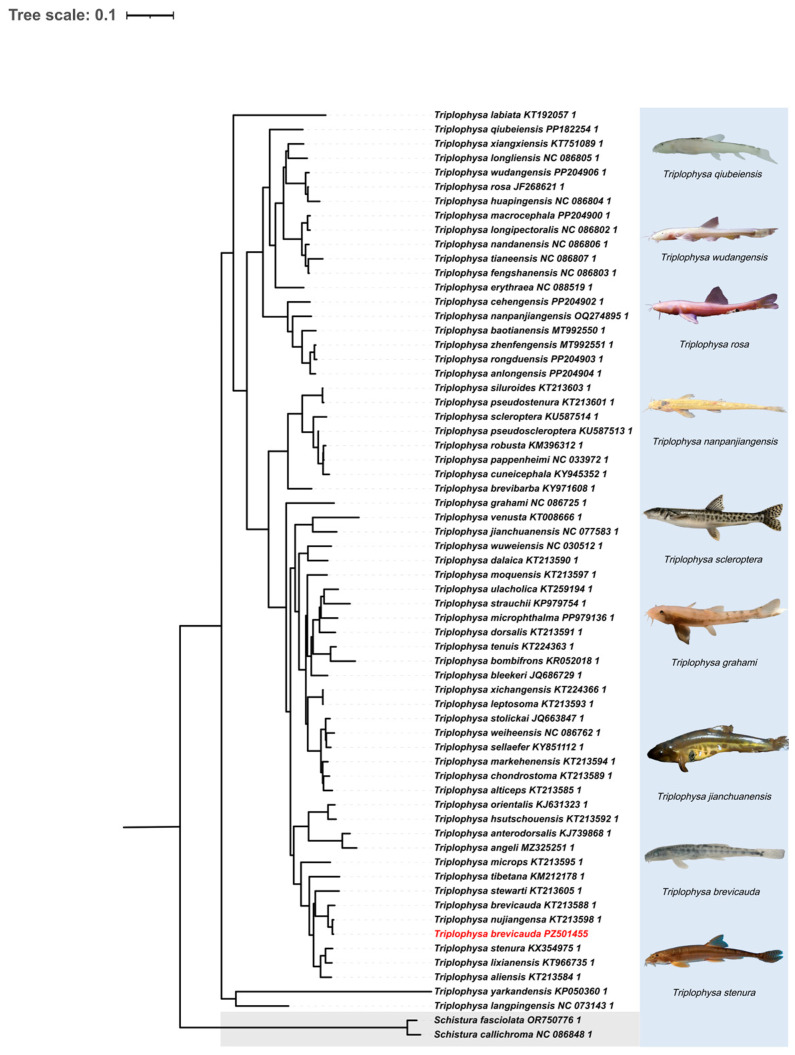
Phylogenetic relationship of 63 *Triplophysa* mitogenomes inferred by ML analyses, based on complete mitochondrial genomes. The number on the branches indicates the maximum likelihood (ML) bootstrap support value. The *T. brevicauda* in this study is marked in red.

To establish the phylogenetic position of *T. brevicauda* within *Triplophysa*, a maximum likelihood (ML) tree was inferred from complete mitochondrial genome sequences combined with published mitogenomes obtained from GenBank. The analysis recovered a fully resolved topology in which all major nodes received bootstrap support ≥0.9. Every sampled *Triplophysa* species formed a strongly supported monophyletic assemblage (BS = 1.00). *T. brevicauda* was resolved as the sister species to *T. nujiangensis* with maximal support (BS = 1.00); this pair, in turn, clustered with *T. stewarti*, *T. stenura*, *T. lixianensis*, *T. aliensis*, *T. tibetana* and *T. microps* in a well-supported clade (BS = 1.00) located mid-tree. The earliest diverging lineages included *T. labiata, T. yarkandensis* and *T. langpingensis*. Notably, *T. brevicauda* did not group with morphologically similar species such as *T. microps*; instead, its clade was positioned adjacent to a predominantly Qinghai-Plateau radiation that includes *T. tibetana* and *T. aliensis* (Figure 2). The topological structure of the Bayesian inference (BI) tree was completely identical to the ML tree, with no discrepancies observed across all branches and clades (Figure S3).

### 3.4. Selective Pressure Analyses

**Figure 3:**
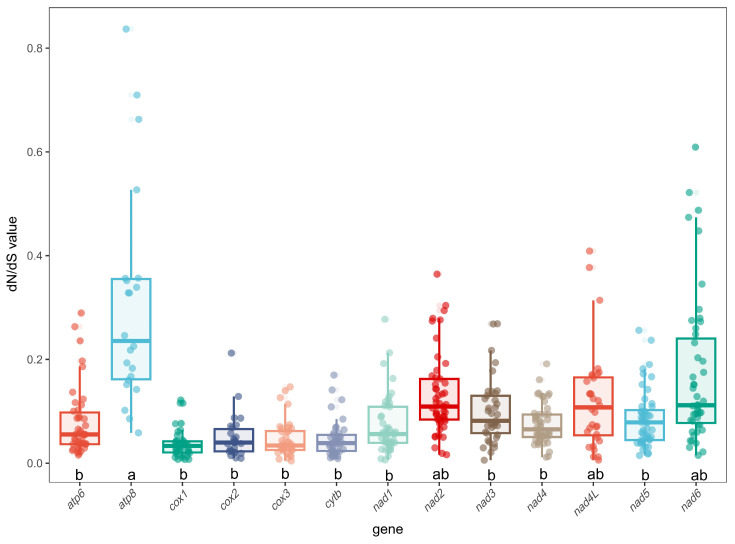
Boxplot of *dN*/*dS* of the 13 PCGs for 63 *Triplophysa* species. These data were calculated with the free ratio model. The distribution of *dN*/*dS* values across genes is depicted, with significance-based groupings indicated. Lowercase letters (a, b, ab) under boxes represent Tukey’s HSD statistical groupings; groups sharing identical letters are not significantly different at *p* < 0.05.

**Figure 4:**
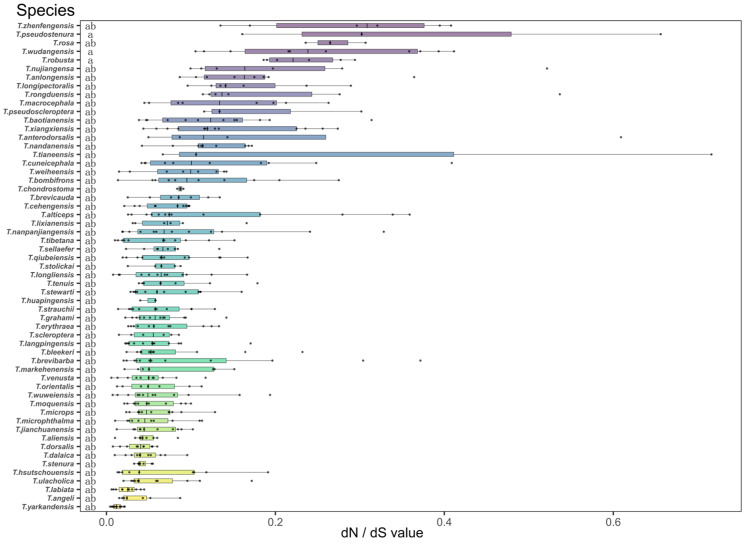
Boxplot of dN/dS values for 63 *Triplophysa* species, showing interspecific variation. Analyses were conducted by using the free ratio model. Lowercase letters (a, ab) under boxes represent Tukey’s HSD statistical groupings; groups sharing identical letters are not significantly different at *p* < 0.05.

The single-ratio model yielded ω = *dN*/*dS* < 1 (range from 0.01 to 0.84) for all genes, indicating pervasive purifying selection. The gene *atp8* exhibited a significantly higher ω than any other locus (*p* < 0.05), except for *nad2*, *nad4L* and *nad6* (Figure 3). Similarly, species-level ω estimates across *Triplophysa* ranged from 0.01 to 0.84, with the vast majority below 0.2, corroborating genus-wide purifying selection (Figure 4).

One-way ANOVA revealed that species differed significantly at the overall level (F = 2.53, df = 55, *p* < 0.001). However, Tukey HSD post hoc comparisons showed that only a small number of species pairs reached a significant level of difference (Table S4). Finally, site model analysis detected three positively selected sites in *nad2* and *nad5*: sites 85 and 318 in *nad2*, and site 33 in *nad5* (Table 1).

We further adopted aBSREL and MEME algorithms in Datamonkey 2.0 to assess episodic selection across the mitochondrial genomes. The aBSREL analysis identified multiple phylogenetic branches of *cox2*, *nad2* and *nad5* under significant branch-specific episodic positive selection. Meanwhile, the MEME test uncovered a number of codon sites subjected to episodic diversifying selection among seven mitochondrial protein-coding genes. All associated results were statistically significant at *p* < 0.05 (Table 2 and Table 3).

## 4. Discussion

### 4.1. Mitochondrial Genome Features

The mitogenome of *T. brevicauda* aligns with published *Triplophysa* sequences [44,45,46,47,48,49,50,51,52,53,54,55,56], spanning 16,570 bp and comprising 13 PCGs, 22 transfer RNAs (tRNAs), two ribosomal RNAs (rRNAs), and a 744 bp D-loop region with an AT content of 56.25%. Notably, this D-loop sequence is remarkably shorter than the average length of approximately 917 bp observed in most other *Triplophysa* species. As a highly variable non-coding region, the mitochondrial control region is a vital molecular marker for aquatic organisms, and its length polymorphism is primarily caused by tandem repeat variations and replication slippage [57]. Such a unique structural trait has been further fixed by long-term geographical isolation and genetic drift in fragmented plateau river systems. In addition, the distinct length difference in D-loop can act as a useful auxiliary characteristic for species identification and phylogenetic analysis within this genus. Considering the high sensitivity of the control region to environmental changes, future investigations on its sequence variation will also help reveal the evolutionary responses of plateau loaches to ongoing climate warming and rising water temperature. Structural variation is confined to the control region and adjacent genes, indicating subtle yet detectable intra-generic divergence. Codon usage is consistent with congeners, with leucine (Leu) > isoleucine (Ile) > phenylalanine (Phe) predominance; ATG is the primary start codon, and TAA serves as the dominant stop codon. Strand gene distribution is conserved, with 28 genes located on the H-strand and nine on the L-strand. Collectively, these features affirm the structural stability of *Triplophysa* mitogenomes.

### 4.2. Phylogenetic Relationship of Triplophysa Analyses

*Triplophysa*, a genus of small benthic fish endemic to the Qinghai-Xizang Plateau and adjacent drainages, represents the most species-rich freshwater fish radiation in this region. Its diversification was closely linked to the episodic uplift of the plateau [14]. Intense geological disturbances and heterogeneous high-altitude environments promoted rapid adaptive radiation, resulting in widespread incomplete lineage sorting and morphologically conserved traits that complicate phylogenetic resolution [58,59].

Previous studies consistently resolve *Triplophysa* into several well-supported major clades [49,60,61,62], yet the identity of the most basal lineage remains uncertain: *T. rosa* when third-codon positions are excluded [14], *T. yarkandensis* based solely on *cox1* data [8], and *T. langpingensis* when using a complete mitochondrial genome dataset—a result congruent with the topology presented here [49]. Such inconsistent phylogenetic results are largely attributed to discrepancies in analytical methodologies adopted by different researchers. Variations in molecular markers, sequence alignment strategies, nucleotide substitution models, tree-building algorithms and outgroup selection all contribute to topological fluctuations. Even differences in data filtering criteria and phylogenetic software can further amplify the divergence of final phylogenetic topologies among independent studies. This discordance likely arises from differences in outgroup selection [63], the effects of rapid radiation [15], and variations in gene sampling and taxon density. Notably, *T. brevicauda* does not group with morphologically similar congeners such as *T. stenura* or *T. lixianensis*, suggesting either morphological convergence or limited diagnostic utility of external characters. By integrating all available mitogenomic data, this study refines the phylogeny of *Triplophysa*, establishes *T. brevicauda* as sister to *T. nujiangensis*, and provides robust molecular evidence for ongoing diversification and adaptive radiation across the Qinghai-Xizang Plateau.

### 4.3. Selective Pressure Analyses

To cope with the complex and variable plateau environment, *Triplophysa* fishes have evolved a suite of adaptive mechanisms, including specialized body coloration [64], enhanced salt stress tolerance [65], and modifications to the visual system [66]. Mitochondria, serving as central hubs of energy metabolism, generate ATP via oxidative phosphorylation and also exhibit gene-level evolutionary signatures associated with hypoxia and cold tolerance in high-altitude fish, providing a critical molecular foundation for environmental adaptation. Notably, the 13 protein-coding genes within the mitochondrial genome encode essential subunits of the respiratory chain and have been extensively studied in this context [67,68].

All 13 mitochondrial protein-coding genes predominantly evolve under strong purifying selection (ω < 1), consistent with the general functional stability required for core oxidative phosphorylation. However, selection intensity varies across genes: *atp8* exhibits the highest ω value, suggesting that its peripheral location in the ATP synthase complex allows limited non-synonymous substitutions while potentially serving as a reservoir for plateau-specific adaptations. Moderately elevated yet constrained evolutionary rates in *nad2*, *nad4L*, and *nad6* imply regulatory flexibility in respiratory chain efficiency under hypoxic or low-temperature conditions. In contrast, cytochrome *c* oxidase subunits (*cox1*, *cox2*, *cox3*) and core NADH dehydrogenase subunits (*nad1*, *nad3*, *nad5*) display minimal non-synonymous divergence, underscoring their indispensable roles in electron transfer. This hierarchical pattern of selective constraint mirrors functional indispensability and may underlie the metabolic remodeling that accompanied the long-term adaptation of *Triplophysa* to the Qinghai-Xizang Plateau.

At the species level, PCGs in *Triplophysa* are predominantly subject to strong purifying selection (ω < 0.2 in most taxa), consistent with the pervasive purifying selection revealed above and confirming an absence of widespread positive selection across the mitogenome. This pattern suggests that maintaining respiratory chain stability has been the primary evolutionary imperative during the genus’ radiation across the Qinghai-Xizang Plateau. Nevertheless, significant interspecific variation in ω values indicates that distinct lineages experience divergent selective pressures, likely reflecting fine-scale adaptation to local ecological gradients—such as altitude, water temperature, and dissolved oxygen levels—or differing life history strategies.

Despite the dominant purifying selection, detections of positively selected sites were found in multiple mitochondrial genes. The relevant genes and corresponding sites are as follows: *cox3* residue 81, a helical structure associated with transmembrane function (Complex III); this transmembrane domain mediates electron transport and proton translocation, and may facilitate adaptation to low dissolved oxygen at high altitudes. *nad4* residue 85, a helical site involved in transmembrane activity; the domain maintains the structural integrity of respiratory chain complex I and participates in proton pumping, possibly linked to energy metabolism under cold and hypoxic conditions. *nad4L* residue 7, another helical site related to transmembrane structure; it stabilizes the assembly of complex I subunits and sustains normal mitochondrial respiration, which is conducive to survival in extreme high-altitude aquatic environments. Thus, adaptation to extreme high-altitude environments appears to occur primarily through targeted functional shifts at a small number of critical residues, rather than genome-wide pervasive positive selection. Ongoing climate warming has led to continuous water temperature increase on the plateau, which disturbs mitochondrial energy metabolism and imposes new adaptive pressure on cold-water fish such as *Triplophysa* [69]. These environmental changes are likely to act on the limited functional sites of mitochondrial genes.

Positive selection on mitochondrial protein-coding genes is concentrated in complex I (NADH: ubiquinone oxidoreductase) [70], the principal site of mitochondrial reactive oxygen species (ROS) generation [71]. Previous studies have shown that high-altitude hypoxia increases superoxide production, with complex I identified as the major source of mitochondrial superoxide [14].

Collectively, against a background of strong purifying selection, *Triplophysa* appears to meet the dual challenges of high-altitude energy metabolism and oxidative stress through precise changes at specific residues in *nad2* and *nad5*. This evolutionary strategy parallels findings in other high-altitude vertebrates. In mammals inhabiting elevated regions, accelerated evolution or positive selection has been detected in several ND subunits—particularly *nad2*, *nad4*, and *nad5*—in species such as *Pantholops hodgsonii* [72], the Tibetan horse [73], and *Bos grunniens* [74]. The signal is especially robust for *nad5*: a phylogenomic analysis of Caprini revealed significantly elevated *dN*/*dS* ratios in high-altitude lineages [72], and a large-scale comparative study of 104 vertebrates identified residues 247 (phenylalanine) and 524 (methionine) in *nad5* as the only positively selected sites shared among all high-elevation taxa [67]. These residues reside in functionally critical domains involved in electron transfer or proton translocation, highlighting the adaptive significance of *nad5* under hypobaric hypoxia. The positively selected sites now detected in *Triplophysa* likely represent a convergent mitochondrial signature of adaptation to high-altitude hypoxia.

In conclusion, this study systematically characterized the mitogenome of *T. brevicauda*, revealed its unique structural features including the shortened D-loop, and resolved its phylogenetic placement within *Triplophysa* using large-scale mitogenomic data. Mitochondrial genes of this genus are generally constrained by strong purifying selection, and the targeted amino acid mutations in *nad2* and *nad5* constitute a typical convergent adaptation to extreme plateau environments. Combined with geological changes and ongoing climate warming on the Qinghai-Xizang Plateau, our findings also imply that the compact mitogenome and site-specific adaptive mutations may help plateau loaches cope with rising water temperature and changing dissolved oxygen conditions. As the most diverse freshwater fish group endemic to the plateau, *Triplophysa* still has many unsolved evolutionary questions. Future multi-omics and in vivo functional verification will deepen our understanding of their adaptive radiation. Overall, our mitogenomic data not only supplements the genetic resources of *Triplophysa*, but also offers a valuable case for studying organismal adaptation to extreme alpine environments worldwide.

## Figures and Tables

**Table 1:** Site model analysis for the 13 PCGs in the genus *Triplophysa*.

Gene	Model	−lnL	Model Comparison	2ΔlnL	df	*p*-Value	Parameters	Positive Site (PP ≥ 80%)
*nad2*	M8	18,494.15166	M8 vs. M8a	1.96	1	0.162344813	1.872 1.874	85 G 0.998 ** 318 Q 1.000 **
	M8a	18,495.12779						
*nad5*	M8	27,292.95475	M8 vs. M8a	8E-06	1	0.997743245	1.364	33 D 0.801
	M8a	27,292.95475						

** Sites with posterior probability ≥ 80% under Bayes Empirical Bayes inference.

**Table 2:** Detections of selective pressure on 13 PCGs in the genus *Triplophysa* used aBSREL.

Gene	Brunch	LRT	Test *p*-Value	ω Distribution Over Sites
*cox2*	*Triplophysa robusta*	35.5	0	ω_1_ = 0.6417, ω_2_ = 675.53
*nad2*	*Triplophysa brevibarba*	91.82	0	ω_1_ = 0.36, ω_2_ = 332.06
	*Triplophysa qiubeiensis*	4.97	0.03008	ω_1_ = 0.11, ω_2_ = 24.99
*nad5*	*Triplophysa xiangxiensis*	12.26	0.00075	ω_1_ = 0.13, ω_2_ = 11.30

**Table 3:** Detections of positively selected sites on 13 PCGs in the genus *Triplophysa* using MEME.

Gene	LRT	*p*-Value	Codon
*atp8*	22.062	0	14
	16.323	0	49
*cox3*	9.599	0.004	81
	4.711	0.044	153
	4.618	0.046	228
*cytb*	7.683	0.01	365
*nad2*	30.578	0	232
*nad4*	4.691	0.044	11
	9.64	0.044	52
	17.623	0	105
	4.948	0.039	283
	14.94	0	460
*nad4l*	7.237	0.012	7
*nad5*	5.929	0.023	1

## Data Availability

The complete mitochondrial genome sequence of *Triplophysa* brevicauda was deposited in GenBank with the accession number PZ501455 (submission ID: SUB16233139). Raw high-throughput sequencing reads were submitted to the NCBI Sequence Read Archive (SRA). All sequence records are currently under routine review and annotation by GenBank staff and will be automatically released to the public database upon completion of processing. Additional datasets supporting the analyses in this study are available in the GitHub repository: https://github.com/wys735/Data.git, accessed on 13 August 2025. All relevant raw data and analytical files can also be obtained from the corresponding author upon reasonable request.

## Supplementary Materials

The following supporting information can be downloaded at https://www.mdpi.com/article/10.3390/genes17070734/s1. Figure S1: Phylogenetic relationships of *Triplophysa* sp. inferred by maximum likelihood (ML) analysis based on the *cytb* gene. Numbers (%) on the branches indicate ML bootstrap support values. Figure S2: Relative synonymous codon usage (RSCU) in the mitochondrial genomes of *T. brevicauda* Figure S3: Phylogenetic relationships of 63 *Triplophysa* mitogenomes inferred by Bayesian inference (BI) analyses, based on 13 PCGs. The number on the branches indicates the Bayesian posterior probability value. The *T. brevicauda* in this study was marked in red. Table S1: Detailed information on the 217 *Triplophysa* and one *Schistura* cytochrome b (*cytb*) gene sequences used for species identification. All sequences were retrieved from NCBI GenBank. Table S2: Detailed information of the mitogenome sequences from 63 *Triplophysa* and two *Schistura* species in this study. All sequences were retrieved from NCBI GenBank. Table S3: Analysis of mitochondrial genome characteristics of *T. brevicauda.* Table S4: Results of one-way ANOVA and Tukey’s HSD post hoc tests for morphometric differentiation among *Triplophysa* species.
