# Supplementary material for: The Complete Mitochondrial Genome of Triplophysa brevicauda and the Analysis of Phylogeny and Selective Pressure Within Genus Triplophysa
Source: Genes (Basel). 2026 Jun 25;17(7):734. doi: 10.3390/genes17070734 (PMC13408864; doi:10.3390/genes17070734)

Figure S1. Phylogenetic relationships of Triplophysa sp. inferred by maximum likelihood (ML) analysis based on the cytb gene. Numbers (%) on the branches indicate ML bootstrap support values.

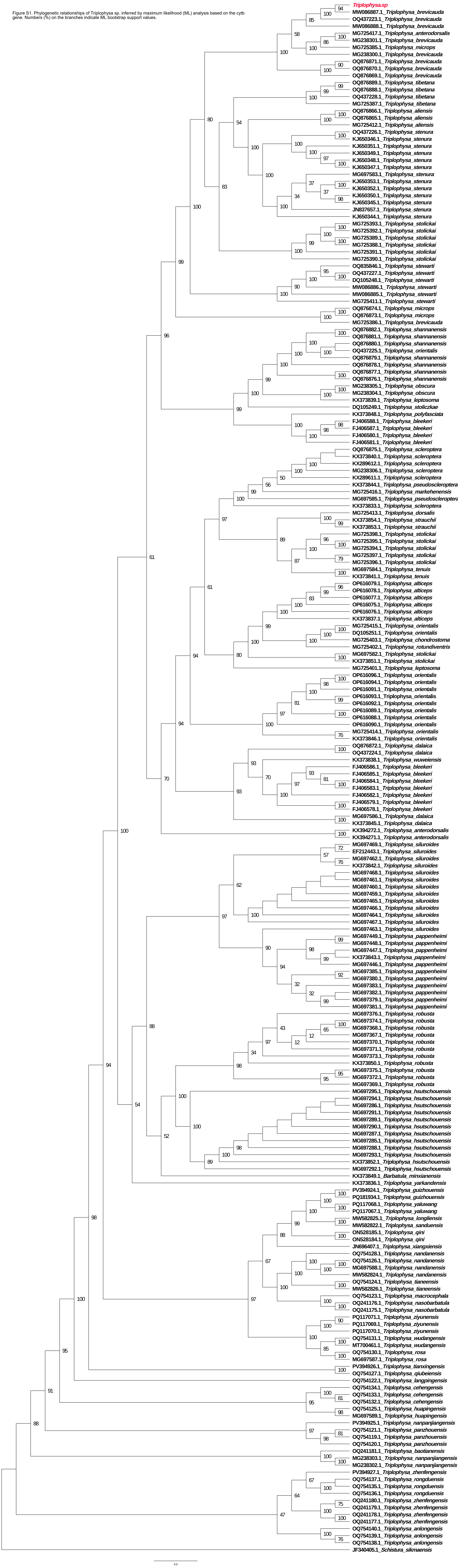

Supplement: Supplementary file 1 [file genes-17-00734-s001.zip › Fig S1-edited.pdf]
