# Supplementary material for: The Complete Mitochondrial Genome of Triplophysa brevicauda and the Analysis of Phylogeny and Selective Pressure Within Genus Triplophysa
Source: Genes (Basel). 2026 Jun 25;17(7):734. doi: 10.3390/genes17070734 (PMC13408864; doi:10.3390/genes17070734)
Supplement: Supplementary file 1 [file genes-17-00734-s001.zip › Fig S3-edited.pdf]

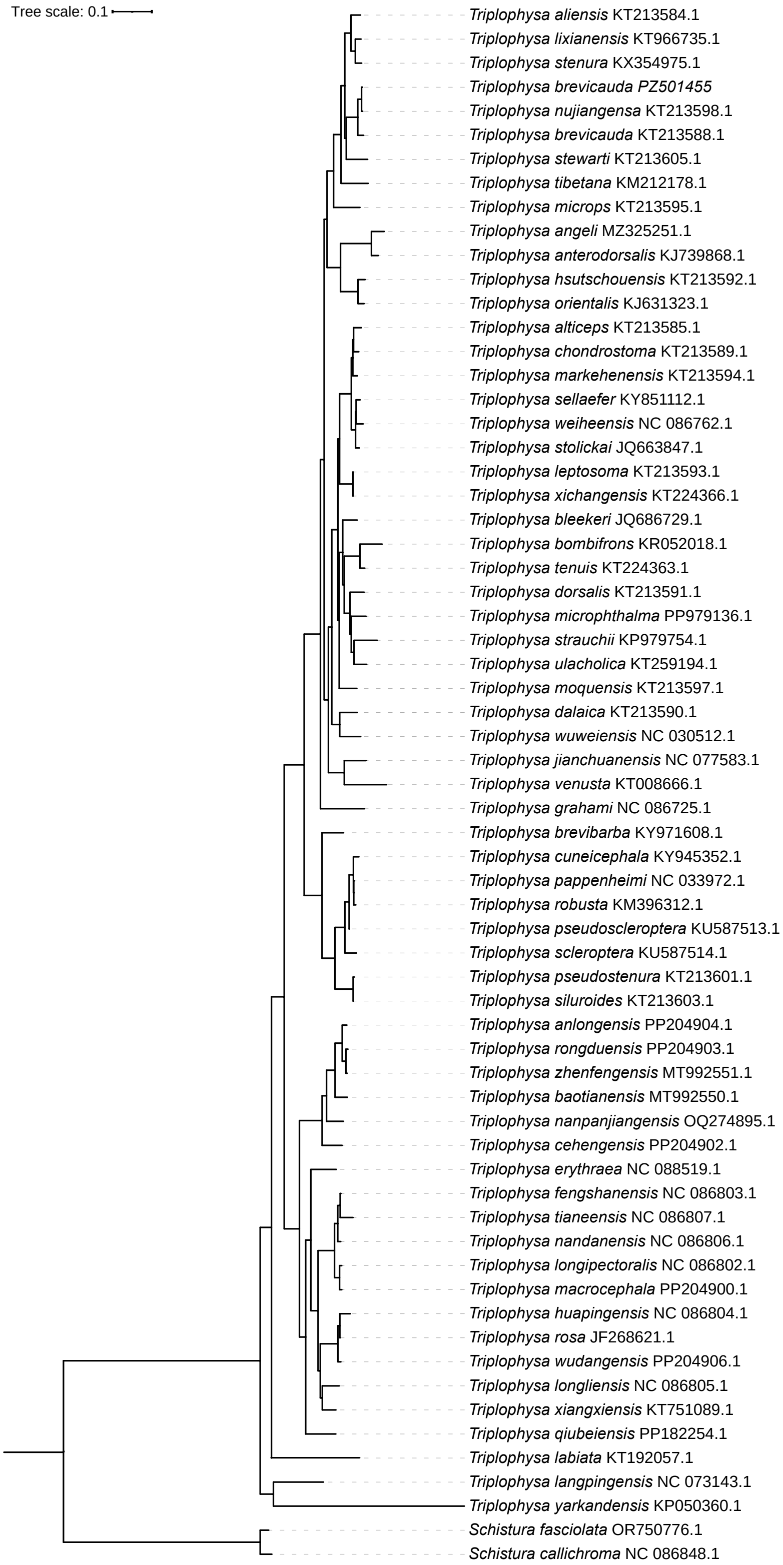

Figure S3: Phylogenetic relationships of 63 Triplophysa mitogenomes inferred by Bayesian inference (BI) analyses, based on 13 PCGs. The number on the branches indicates the Bayesian posterior probability value.
