# Supplementary material for: The Complete Mitochondrial Genome of Triplophysa brevicauda and the Analysis of Phylogeny and Selective Pressure Within Genus Triplophysa
Source: Genes (Basel). 2026 Jun 25;17(7):734. doi: 10.3390/genes17070734 (PMC13408864; doi:10.3390/genes17070734)
Supplement: Supplementary file 1 [file genes-17-00734-s001.zip › Table S1 - edited.pdf]

Table S1. Detailed information on the 217 *Triplophysa* and one *Schistura* cytochrome b (*cytb*) gene sequences used for species identification. All sequences were retrieved from NCBI GenBank.

Species	Accession No
Triplophysa zhenfengensis	PV394927.1
Triplophysa tianxingensis	PV394926.1
Triplophysa nanpanjiangensis	PV394925.1
Triplophysa guizhouensis	PV394924.1
Triplophysa guizhouensis	PQ181934.1
Triplophysa ziyunensis	PQ117071.1
Triplophysa ziyunensis	PQ117070.1
Triplophysa ziyunensis	PQ117069.1
Triplophysa yaluwang	PQ117068.1
Triplophysa yaluwang	PQ117067.1
Triplophysa tibetana	OQ876889.1
Triplophysa tibetana	OQ876888.1
Triplophysa shannanensis	OQ876882.1
Triplophysa shannanensis	OQ876881.1
Triplophysa shannanensis	OQ876880.1
Triplophysa shannanensis	OQ876879.1
Triplophysa shannanensis	OQ876878.1
Triplophysa shannanensis	OQ876877.1

Triplophysa shannanensis	OQ876876.1
Triplophysa scleroptera	OQ876875.1
Triplophysa microps	OQ876874.1
Triplophysa microps	OQ876873.1
Triplophysa dalaica	OQ876872.1
Triplophysa brevicauda	OQ876871.1
Triplophysa brevicauda	OQ876870.1
Triplophysa brevicauda	OQ876869.1
Triplophysa aliensis	OQ876866.1
Triplophysa aliensis	OQ876865.1
Triplophysa stewarti	OQ835846.1
Triplophysa anlongensis	OQ754140.1
Triplophysa anlongensis	OQ754139.1
Triplophysa anlongensis	OQ754138.1
Triplophysa rongduensis	OQ754137.1
Triplophysa rongduensis	OQ754136.1
Triplophysa rongduensis	OQ754135.1
Triplophysa cehengensis	OQ754134.1
Triplophysa cehengensis	OQ754133.1
Triplophysa cehengensis	OQ754132.1
Triplophysa wudangensis	OQ754131.1
Triplophysa rosa	OQ754130.1

Triplophysa nandanensis	OQ754128.1
Triplophysa qiubeiensis	OQ754127.1
Triplophysa nandanensis	OQ754126.1
Triplophysa huapingensis	OQ754125.1
Triplophysa tianeensis	OQ754124.1
Triplophysa macrocephala	OQ754123.1
Triplophysa langpingensis	OQ754122.1
Triplophysa panzhouensis	OQ754121.1
Triplophysa panzhouensis	OQ754120.1
Triplophysa panzhouensis	OQ754119.1
Triplophysa tibetana	OQ437228.1
Triplophysa stewarti	OQ437227.1
Triplophysa stenura	OQ437226.1
Triplophysa orientalis	OQ437225.1
Triplophysa dalaica	OQ437224.1
Triplophysa brevicauda	OQ437223.1
Triplophysa baotianensis	OQ241181.1
Triplophysa zhenfengensis	OQ241180.1
Triplophysa zhenfengensis	OQ241179.1
Triplophysa zhenfengensis	OQ241178.1
Triplophysa zhenfengensis	OQ241177.1
Triplophysa nasobarbatula	OQ241176.1

Triplophysa nasobarbatula	OQ241175.1
Triplophysa orientalis	OP616096.1
Triplophysa orientalis	OP616094.1
Triplophysa orientalis	OP616093.1
Triplophysa orientalis	OP616092.1
Triplophysa orientalis	OP616091.1
Triplophysa orientalis	OP616090.1
Triplophysa orientalis	OP616089.1
Triplophysa orientalis	OP616088.1
Triplophysa alticeps	OP616079.1
Triplophysa alticeps	OP616078.1
Triplophysa alticeps	OP616077.1
Triplophysa alticeps	OP616076.1
Triplophysa alticeps	OP616075.1
Triplophysa qini	ON528185.1
Triplophysa qini	ON528184.1
Triplophysa tianeensis	MW582826.1
Triplophysa longliensis	MW582825.1
Triplophysa nandanensis	MW582824.1
Triplophysa sanduensis	MW582822.1
Triplophysa brevicauda	MW086888.1
Triplophysa brevicauda	MW086887.1
Triplophysa stewarti	MW086886.1
Triplophysa stewarti	MW086885.1
Triplophysa wudangensis	MT700461.1
Triplophysa anterodorsalis	MG725417.1
Triplophysa markehenensis	MG725416.1

Triplophysa orientalis	MG725415.1
Triplophysa orientalis	MG725414.1
Triplophysa dorsalis	MG725413.1
Triplophysa aliensis	MG725412.1
Triplophysa stewarti	MG725411.1
Triplophysa chondrostoma	MG725403.1
Triplophysa rotundiventris	MG725402.1
Triplophysa leptosoma	MG725401.1
Triplophysa stolickai	MG725398.1
Triplophysa stolickai	MG725397.1
Triplophysa stolickai	MG725396.1
Triplophysa stolickai	MG725395.1
Triplophysa stolickai	MG725394.1
Triplophysa stolickai	MG725393.1
Triplophysa stolickai	MG725392.1
Triplophysa stolickai	MG725391.1
Triplophysa stolickai	MG725390.1
Triplophysa stolickai	MG725389.1
Triplophysa stolickai	MG725388.1
Triplophysa tibetana	MG725387.1
Triplophysa brevicauda	MG725386.1
Triplophysa microps	MG725385.1
Triplophysa huapingensis	MG697589.1
Triplophysa nandanensis	MG697588.1
Triplophysa rosa	MG697587.1
Triplophysa dalaica	MG697586.1
Triplophysa pseudoscleroptera	MG697585.1
Triplophysa tenuis	MG697584.1

Triplophysa stenura	MG697583.1
Triplophysa stolickai	MG697582.1
Triplophysa siluroides	MG697469.1
Triplophysa siluroides	MG697468.1
Triplophysa siluroides	MG697467.1
Triplophysa siluroides	MG697466.1
Triplophysa siluroides	MG697465.1
Triplophysa siluroides	MG697464.1
Triplophysa siluroides	MG697463.1
Triplophysa siluroides	MG697462.1
Triplophysa siluroides	MG697461.1
Triplophysa siluroides	MG697460.1
Triplophysa siluroides	MG697459.1
Triplophysa pappenheimi	MG697449.1
Triplophysa pappenheimi	MG697448.1
Triplophysa pappenheimi	MG697447.1
Triplophysa pappenheimi	MG697446.1
Triplophysa pappenheimi	MG697385.1
Triplophysa pappenheimi	MG697383.1
Triplophysa pappenheimi	MG697382.1
Triplophysa pappenheimi	MG697381.1
Triplophysa pappenheimi	MG697380.1
Triplophysa pappenheimi	MG697379.1
Triplophysa robusta	MG697376.1
Triplophysa robusta	MG697375.1
Triplophysa robusta	MG697374.1
Triplophysa robusta	MG697373.1
Triplophysa robusta	MG697372.1

Triplophysa robusta	MG697371.1
Triplophysa robusta	MG697370.1
Triplophysa robusta	MG697369.1
Triplophysa robusta	MG697368.1
Triplophysa robusta	MG697367.1
Triplophysa hsutschouensis	MG697295.1
Triplophysa hsutschouensis	MG697294.1
Triplophysa hsutschouensis	MG697293.1
Triplophysa hsutschouensis	MG697292.1
Triplophysa hsutschouensis	MG697291.1
Triplophysa hsutschouensis	MG697290.1
Triplophysa hsutschouensis	MG697289.1
Triplophysa hsutschouensis	MG697288.1
Triplophysa hsutschouensis	MG697287.1
Triplophysa hsutschouensis	MG697286.1
Triplophysa hsutschouensis	MG697285.1
Triplophysa scleroptera	MG238306.1
Triplophysa obscura	MG238305.1
Triplophysa obscura	MG238304.1
Triplophysa nanpanjiangensis	MG238303.1
Triplophysa nanpanjiangensis	MG238302.1
Triplophysa brevicauda	MG238301.1
Triplophysa brevicauda	MG238300.1
Triplophysa anterodorsalis	KX394272.1
Triplophysa anterodorsalis	KX394271.1
Triplophysa strauchii	KX373854.1
Triplophysa strauchii	KX373853.1
Triplophysa hsutschouensis	KX373852.1

Triplophysa stolickai	KX373851.1
Triplophysa robusta	KX373850.1
Triplophysa polyfascia	KX373848.1
Triplophysa orientalis	KX373846.1
Triplophysa dalaica	KX373845.1
Triplophysa pseudoscleroptera	KX373844.1
Triplophysa pappenheimi	KX373843.1
Triplophysa siluroides	KX373842.1
Triplophysa tenuis	KX373841.1
Triplophysa scleroptera	KX373840.1
Triplophysa leptosoma	KX373839.1
Triplophysa wuweiensis	KX373838.1
Triplophysa alticeps	KX373837.1
Triplophysa yarkandensis	KX373836.1
Triplophysa scleroptera	KX373833.1
Triplophysa scleroptera	KX289612.1
Triplophysa scleroptera	KX289611.1
Triplophysa stenura	KJ650353.1
Triplophysa stenura	KJ650352.1
Triplophysa stenura	KJ650351.1
Triplophysa stenura	KJ650350.1
Triplophysa stenura	KJ650349.1
Triplophysa stenura	KJ650348.1
Triplophysa stenura	KJ650347.1
Triplophysa stenura	KJ650346.1
Triplophysa stenura	KJ650345.1
Triplophysa stenura	KJ650344.1
Triplophysa stenura	JN837657.1

Triplophysa xiangxiensis	JN696407.1
Triplophysa bleekeri	FJ406588.1
Triplophysa bleekeri	FJ406587.1
Triplophysa bleekeri	FJ406586.1
Triplophysa bleekeri	FJ406585.1
Triplophysa bleekeri	FJ406584.1
Triplophysa bleekeri	FJ406583.1
Triplophysa bleekeri	FJ406582.1
Triplophysa bleekeri	FJ406581.1
Triplophysa bleekeri	FJ406580.1
Triplophysa bleekeri	FJ406579.1
Triplophysa bleekeri	FJ406578.1
Triplophysa siluroides	EF212443.1
Triplophysa orientalis	DQ105251.1
Triplophysa stoliczkae	DQ105249.1
Triplophysa stewarti	DQ105248.1
Schistura.sikmaensis	JF340405.1
Barbatula minxianensis	KX373849.1
