# Supplementary material for: The Complete Mitochondrial Genome of Triplophysa brevicauda and the Analysis of Phylogeny and Selective Pressure Within Genus Triplophysa
Source: Genes (Basel). 2026 Jun 25;17(7):734. doi: 10.3390/genes17070734 (PMC13408864; doi:10.3390/genes17070734)
Supplement: Supplementary file 1 [file genes-17-00734-s001.zip › Table S2 - edited.pdf]

Table S2. Detailed information of the mitogenome sequences from 63 *Triplophysa* and two *Schistura* species in this study. All sequences were retrieved from NCBI GenBank

Species	Size(bp)	Accession No
T.microphthalma	16569	PP979136.1
T.wudangensis	16557	PP204906.1
T.anlongensis	16562	PP204904.1
T.rongduensis	16564	PP204903.1
T.cehengensis	16562	PP204902.1
T.macrocephala	16606	PP204900.1
T.qiubeiensis	16585	PP182254.1
T.nanpanjiangensis	16558	OQ274895.1
T.erythraea	16565	NC_088519.1
T.tianeensis	16573	NC_086807.1
T.nandanensis	16604	NC_086806.1
T.longliensis	16570	NC_086805.1
T.huapingensis	16570	NC_086804.1
T.fengshanensis	16607	NC_086803.1
T.longipectoralis	16609	NC_086802.1
T.weiheensis	16570	NC_086762.1
T.grahami	16566	NC_086725.1
T.jianchuanensis	16569	NC_077583.1

T.langpingensis	16567	NC_073143.1
T.pappenheimi	16572	NC_033972.1
T.wuweiensis	16681	NC_030512.1
T.angeli	16569	MZ325251.1
T.zhenfengensis	16564	MT992551.1
T.baotianensis	16576	MT992550.1
T.brevibarba	16570	KY971608.1
T.cuneicephala	16571	KY945352.1
T.sellaefer	16571	KY851112.1
T.stenura	16569	KX354975.1
T.scleroptera	16574	KU587514.1
T.pseudoscleroptera	16574	KU587513.1
T.lixianensis	16570	KT966735.1
T.xiangxiensis	16598	KT751089.1
T.ulacholica	16568	KT259194.1
T.xichangensis	16570	KT224366.1
T.tenuis	16571	KT224363.1
T.stewarti	16567	KT213605.1
T.siluroides	16574	KT213603.1
T.pseudostenura	16638	KT213601.1
T.nujiangensa	16570	KT213598.1
T.moquensis	16571	KT213597.1

T.microps	16571	KT213595.1
T.markehenensis	16569	KT213594.1
T.leptosoma	16570	KT213593.1
T.hsutschouensis	16571	KT213592.1
T.dorsalis	16572	KT213591.1
T.dalaica	16576	KT213590.1
T.chondrostoma	16568	KT213589.1
T.brevicauda	16572	KT213588.1
T.alticeps	16572	KT213585.1
T.aliensis	16565	KT213584.1
T.labiata	16584	KT192057.1
T.venusta	16574	KT008666.1
T.bombifrons	16569	KR052018.1
T.strauchii	16568	KP979754.1
T.yarkandensis	16574	KP050360.1
T.robusta	16572	KM396312.1
T.tibetana	16571	KM212178.1
T.anterodorsalis	16567	KJ739868.1
T.orientalis	16562	KJ631323.1
T.bleekeri	16573	JQ686729.1
T.stolickai	16571	JQ663847.1
T.rosa	16585	JF268621.1

S.callichroma	16574	NC_086848.1
S.fasciolata	16588	OR750776.1
