# Supplementary material for: The Complete Mitochondrial Genome of Triplophysa brevicauda and the Analysis of Phylogeny and Selective Pressure Within Genus Triplophysa
Source: Genes (Basel). 2026 Jun 25;17(7):734. doi: 10.3390/genes17070734 (PMC13408864; doi:10.3390/genes17070734)
Supplement: Supplementary file 1 [file genes-17-00734-s001.zip › Table S3 - edited.pdf]

**Table S3**. Analysis of mitochondrial genome characteristics of *T. brevicauda*

| **Genes** | **Start** | **Stop** | **Size(bp)** | **Start Coding** | **Stop Coding** | **Strand** |
|---|---|---|---|---|---|---|
| *trnL2(taa)* | 1 | 75 | 75 | | | + |
| *rrnL* | 76 | 1733 | 1658 | | | + |
| *trnV(tac)* | 1754 | 1825 | 72 | | | + |
| *rrnS* | 1828 | 2775 | 948 | | | + |
| *trnF(gaa)* | 2776 | 2844 | 69 | | | + |
| *OH* | 3013 | 3756 | 744 | | | - |
| *trnP(tgg)* | 3762 | 3831 | 70 | | | - |
| *trnT(tgt)* | 3830 | 3900 | 71 | | | + |
| *cytb* | 3901 | 5041 | 1141 | ATG | T | + |
| *trnE(ttc)* | 5046 | 5114 | 69 | | | - |
| *nad6* | 5115 | 5636 | 522 | ATG | TAG | - |
| *nad5* | 5633 | 7471 | 1839 | ATG | TAA | + |
| *trnL1(tag)* | 7472 | 7544 | 73 | | | + |
| *trnS1(gct)* | 7546 | 7613 | 68 | | | + |
| *trnH(gtg)* | 7614 | 7683 | 70 | | | + |
| *nad4* | 7691 | 9065 | 1375 | ATG | T | + |
| *nad4L* | 9059 | 9355 | 297 | ATG | TAA | + |
| *trnR(tcg)* | 9356 | 9425 | 70 | | | + |
| *nad3* | 9424 | 9774 | 351 | ATG | TAG | + |
| *trnG(tcc)* | 9775 | 9847 | 73 | | | + |

| | | | | | | |
|---|---|---|---|---|---|---|
| *cox3* | 9847 | 10631 | 785 | ATG | TA | + |
| *atp6* | 10631 | 11314 | 684 | ATG | TAA | + |
| *atp8* | 11305 | 11472 | 168 | ATG | TAA | + |
| *trnK(ttt)* | 11474 | 11549 | 76 | | | + |
| *cox2* | 11574 | 12261 | 688 | ATG | T | + |
| *trnD(gtc)* | 12254 | 12326 | 73 | | | + |
| *trnS2(tga)* | 12329 | 12399 | 71 | | | - |
| *cox1* | 12400 | 13950 | 1551 | GTG | TAA | + |
| *trnY(gta)* | 13952 | 14020 | 69 | | | - |
| *trnC(gca)* | 14021 | 14086 | 66 | | | - |
| *OL* | 14085 | 14115 | 31 | | | + |
| *trnN(gtt)* | 14118 | 14190 | 73 | | | - |
| *trnA(tgc)* | 14192 | 14260 | 69 | | | - |
| *trnW(tca)* | 14263 | 14332 | 70 | | | + |
| *nad2* | 14331 | 15377 | 1047 | ATG | TAG | + |
| *trnM(cat)* | 15378 | 15446 | 69 | | | + |
| *trnQ(ttg)* | 15448 | 15518 | 71 | | | - |
| *trnI(gat)* | 15517 | 15588 | 72 | | | + |
| *nad1* | 15596 | 16570 | 975 | ATG | TAA | + |
