# Supplementary figures and images for: The Complete Mitochondrial Genome of Triplophysa brevicauda and the Analysis of Phylogeny and Selective Pressure Within Genus Triplophysa
Source: Genes (Basel). 2026 Jun 25;17(7):734. doi: 10.3390/genes17070734 (PMC13408864; doi:10.3390/genes17070734)

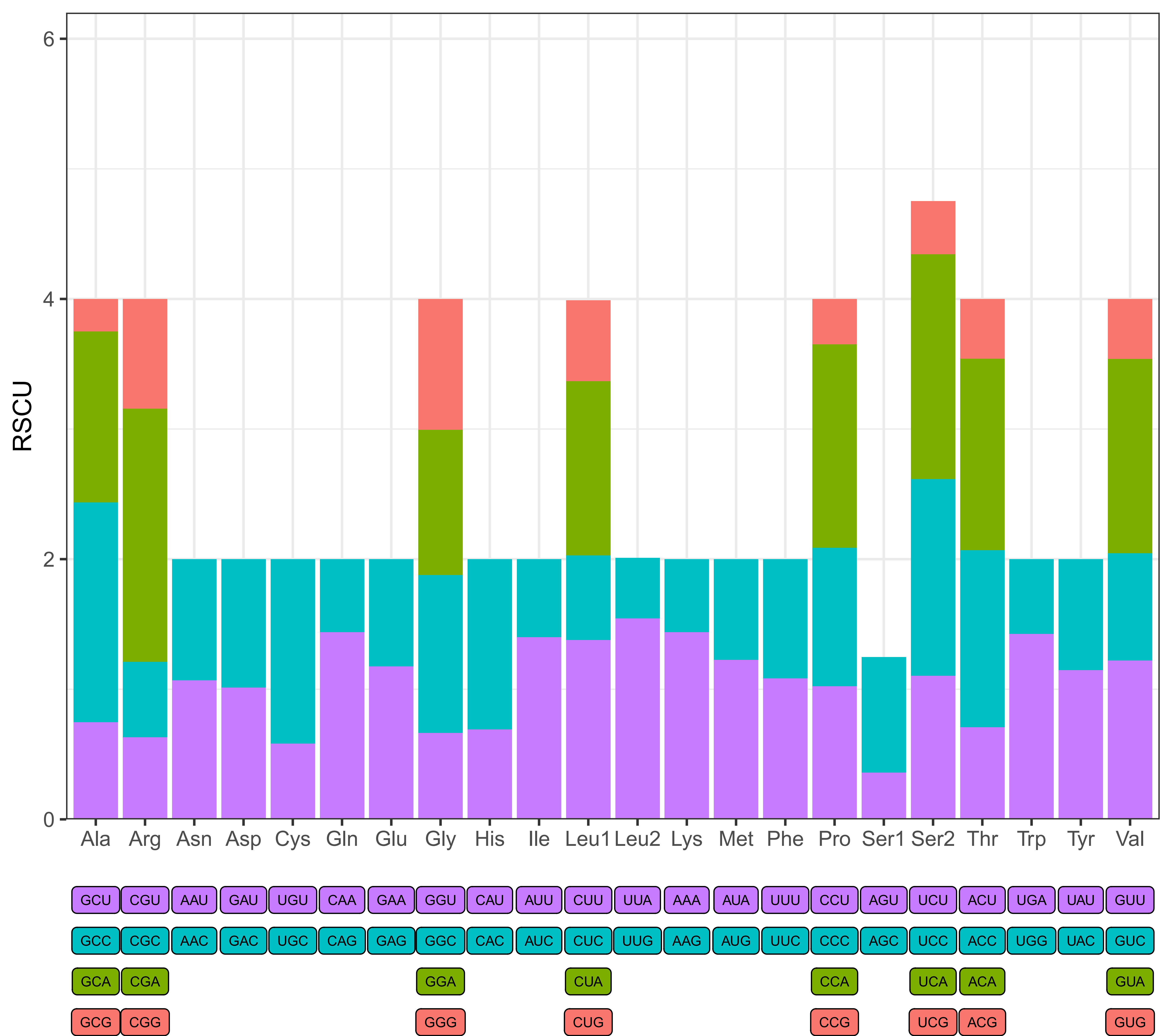

Figure S2. Relative synonymous codon usage (RSCU) in the mitochondrial genomes of *T. brevicauda*.

Supplement: Supplementary file 1 [file genes-17-00734-s001.zip › Fig S2- edited.pdf]
